# Preferences for Depression Treatment Including Internet-Based Interventions: Results From a Large Sample of Primary Care Patients

**DOI:** 10.3389/fpsyt.2018.00181

**Published:** 2018-05-17

**Authors:** Marie Dorow, Margrit Löbner, Alexander Pabst, Janine Stein, Steffi G. Riedel-Heller

**Affiliations:** Institute of Social Medicine, Occupational Health and Public Health, University of Leipzig, Leipzig, Germany

**Keywords:** treatment preferences, depression, primary care, new media, iCBT, e-mental health

## Abstract

**Background:** To date, little is known about treatment preferences for depression concerning new media. This study aims to (1) investigate treatment preferences for depression including internet-based interventions and (2) examine subgroup differences concerning age, gender and severity of depression as well as patient-related factors associated with treatment preferences.

**Methods:** Data were derived from the baseline assessment of the @ktiv-trial. Depression treatment preferences were assessed from *n* = 641 primary care patients with mild to moderate depression regarding the following treatments: medication, psychotherapy, combined treatment, alternative treatment, talking to friends and family, exercise, self-help literature, and internet-based interventions. Depression severity was specified by GPs according to ICD-10 criteria. Ordinal logistic regression models were conducted to identify associated factors of treatment preferences.

**Results:** Patients had a mean age of 43.9 years (*SD* = 13.8) and more than two thirds (68.6%) were female. About 43% of patients had mild depression while 57% were diagnosed with moderate depression. The majority of patients reported strong preferences for psychotherapy, talking to friends and family, and exercise. About one in five patients was very likely to consider internet-based interventions in case of depression. Younger patients expressed significantly stronger treatment preferences for psychotherapy and internet-based interventions than older patients. The most salient factors associated with treatment preferences were the patients' education and perceived self-efficacy.

**Conclusions:** Patients with depression report individually different treatment preferences.Our results underline the importance of shared decision-making within primary care. Future studies should investigate treatment preferences for different types of internet-based interventions.

## Introduction

Depression is a common, but often unrecognized and under-treated condition in primary care (1). According to estimates just about every 10th patient of a general practitioner (GP) seeks help due to depression in Germany (2). In line with this, Jacobi et al. (3) found a high prevalence of depressive syndromes according to ICD-10 criteria (11.3%) in a large sample of primary care patients, confirming similar prevalence rates of other studies (4, 5). Moreover, primary care is faced with a large amount of patients with sub-threshold depression. These patients do not meet the full criteria of a depressive disorder but may still be in need of help to manage their symptoms (3, 6).

Patients' preferences regarding a specific depression treatment may not only influence patients' treatment satisfaction, but may also have major implications for treatment adherence and outcome (7, 8). Thus, Gelhorn et al. conclude in their systematic literature review, that patient preferences are strongly associated with outcomes such as treatment initiation, treatment persistence, engagement and the development of therapeutic alliance. Therefore, the National Disease Management Guideline (S-3-Guideline) for unipolar depression (9) recommends a seven-step model of shared decision-making for health care providers (10, 11) and demands that the assessment and understanding of patient preferences should be considered as an indispensable step within the decision-making process.

Nevertheless, studies regarding treatment preferences for depression as well as associated factors in primary care settings are rare and so far have mainly focused on comparing psychotherapy and pharmacotherapy as preferred treatments (8, 12). This perspective leaves new treatment components like the use of new media aside. Moreover, most studies investigating treatment preferences assess the preference of one treatment toward another (13). However, especially for internet-based interventions there is growing interest in combining internet-based cognitive behavioral therapy (iCBT) with other treatments, such as face-to-face-psychotherapy, in the form of blended care (14, 15). Therefore, assessing the strength of preference for different treatment methods is important in order to offer a broad treatment range. Gaining knowledge about patients' preferences regarding various treatment options for depression including new approaches could enhance individually tailored treatment concepts and may lead to improved management of depression in primary care.

The present study therefore aims to investigate the following research questions:

1. What are treatment preferences for depression in primary care patients?
2. Are there subgroup differences in treatment preferences concerning gender, age and severity of depression?
3. Which sociodemographic, work-related and illness-related variables are associated with different treatment preferences for depression in primary care patients?
4. To what extent are internet-based interventions for depression preferred by primary care patients and which variables are associated with preferences for new media based approaches?

## Materials and methods

### Study design and sample

Data were derived from the @ktiv-trial (trial registration number: DRKS00005075). The trial was approved by the institutional review boards (Ethics Committees) of the University of Leipzig (reference number 222/14ff) and of the Australian National University (reference number 2013/342). The @ktiv-trial consists of a baseline assessment followed by a 6 week and a 6 month follow-up. The present study focuses on the analysis of cross-sectional data from the baseline assessment.

Patients were recruited from 112 primary care practices in Central Germany and had to fulfill the following criteria: (1) age of 18 years and above, (2) positive screening for mild to moderately severe depressive symptoms according to the 9 item version of the Patient Health Questionnaire (PHQ-9) with a total score between 5 and 19 points, (3) mild or moderate depression according to the GP's diagnosis based on ICD-10 criteria (F32.0, F32.1, F33.0, F33.1), (4) German as first language, and (5) home internet access and regular use of the Internet. Patients were excluded in case of (1) severe or persistent depression (ICD-10: F32.2, F32.3, F33.2, F33.3, F34), (2) organic mental disorders (ICD-10: F00-F09), (3) alcohol or drug dependence (F10-F16, F18, F19), (4) schizophrenia and schizoaffective disorders (F20-F29), (5) bipolar disorders (F31), (6) suicidality, (7) fatal somatic disease (e.g., final stadium of cancer), (8) current grief (due to recent loss of a beloved person), and/or (9) receiving psychotherapy at the time of recruitment.

After giving written consent *N* = 647 patients filled in a written self-report questionnaire in the primary care practice for baseline assessment. Recruitment of patients was conducted between February 1, 2014 and August 31, 2015.

### Variables and instruments

The patient self-report questionnaire comprised a wide-ranging set of structured scales and variables.

#### Primary outcome

Treatment preferences were assessed using an adapted 8-item rating scale based on previous research (16, 17) with each item representing a different treatment option for depressive disorders. The treatment options were: medication, psychotherapy, combined treatment (medication and psychotherapy), alternative therapy options such as alternative practitioners, talking to friends and family, exercise, self-help literature and internet-based interventions. Patients were asked to indicate to what extent they would consider the different treatment options in case of depression on a scale ranging from 1 (“very unlikely”) to 5 (“very likely”).

#### Sociodemographic and work-related variables

Sociodemographic data included the patients' age, gender, marital status (married, single, divorced, widowed) and educational level according to the new CASMIN educational classification system (18). For work-related information the patients' vocational qualification was collected.

#### Illness-related variables

As part of the recruitment process GPs were asked to specify the severity of depression, i.e., whether patients had mild or moderate depression according to ICD-10 criteria.

Other illness-related variables were collected from the patients' self-report. Patients were asked to indicate whether they had ever received treatment due to emotional stress (such as sadness, anxiousness or mental overload) before (treatment history; yes/no). Comorbid panic disorder (PD; F41.0 or F40.01) and/or generalized anxiety disorder (GAD; F41.1 or F41.9) according to ICD-10 criteria were assessed with the Patient Health Questionnaire ([PHQ-D; (19)], a validated German self-report version of the Primary Care Evaluation of Mental Disorders [PRIME-MD; (20)]). In addition, the 6-item subscale Hope and Self-efficacy with a minimum of 0 and maximum score of 24 points from the questionnaire for the assessment of Empowerment in Patients with Affective and Schizophrenic disorders [EPAS; (21)] was applied. Health-related quality of life (HRQOL) was measured using the 5-level version of the health state classifier EQ-5D-5L of the EuroQol Group (22). Sum scores of HRQOL had a possible range from 0 and 100 with higher values indicating higher HRQOL.

### Statistical analysis

Patients with missing data on age, gender or the primary outcome, i.e., patients who did not rate any item on treatment preferences, were excluded from analysis. Thus, the analytical sample consists of *n* = 641 patients. Missing data on the determinants ranged from 0.2% (marital status) to 7.8% (comorbid PD) and were replaced using multiple imputation by chained equations (23). We used the pooled estimates of 50 imputed datasets for all analyses.

Descriptive data are presented as mean with standard deviation (SD) or absolute frequencies and percentages. Subgroup analyses were performed for gender, age, and severity of depression. Age was divided into three groups (18–30, 31–50, and 51–82 years) to indicate age-related differences. Subgroup comparisons were evaluated using Wilcoxon two-sample tests or Cuzick's (24) trend test across ordered groups. In order to identify whether treatment preferences are associated with sociodemographic and illness-related variables eight ordinal logistic regression models were conducted, one for each treatment option. All continuous variables in the models were centered (mean = 0; *SD* = 1) to reduce multicollinearity. The proportionality of odds assumption was fulfilled in all ordinal regression models as suggested by approximate likelihood ratio tests of proportionality of odds (25).

All statistical analyses were performed using the Statistical Package for the Social Sciences 24.0 for Windows (SPSS Inc., Chicago, IL) or Stata 13.1 SE (Stata Corp LP, College Station, TX). Given the total number of eight models tested, all analyses are based on a more stringent level of significance with a *p*-value below 0.01.

## Results

### Sample characteristics

Table 1 shows the sociodemographic, work-related and illness-related characteristics of the sample. Study participants had a mean age of 43.9 years (*SD* = 13.8) with the following distribution of age groups: 21.5% (18–30 years), 43.8% (31–50 years) and 34.6% (51–82 years). Regarding older patients, *n* = 35 (5.5%) individuals in our sample were 65+ years of age and *n* = 9 (1.5%) were older than 75 years. More than two thirds (68.6%) of the patients were female. The majority of patients were either married (42.8%) or single (41.2%), had medium education (55.9%) and completed an apprenticeship (57.9%). 43.4% of patients had mild and 56.6% had moderate depression according to their GP. Two in three (67%) patients reported that they had been treated for emotional stress before, about one out of four patients had a comorbid panic disorder (27.0%) and 22.9% had a comorbid generalized anxiety disorder. Mean values for self-efficacy (EPAS) and HRQOL (EQ-5D-5L) were 11.9 (*SD* = 4.5) and 74.4 (*SD* = 13.4), respectively.

**Table 1:** Sample characteristics (*n* = 641).

**SOCIODEMOGRAPHIC AND WORK-RELATED VARIABLES**	
Age, mean (SD)	43.9(13.8)
**Age groups**	
18–30	138(21.5)
31–50	281(43.8)
51–82	222(34.6)
**Gender**	
Female	440(68.6)
Male	201(31.4)
**Marital status**	
Married	274(42.8)
Single	264(41.2)
Divorced/widowed	103(16.1)
**Educational level**	
High	194(30.3)
Middle	358(55.9)
Low	89(13.9)
**Vocational qualification**	
None/other qualification	36(5.6)
Still undergoing vocational training	25(3.9)
Completed apprenticeship	371(57.9)
Secondary vocational education	101(15.8)
University degree	108(16.9)
**ILLNESS-RELATED VARIABLES**	
**Depression diagnosis by GP**	
Mild depression	278(43.4)
Moderate depression	363(56.6)
Treatment history	432(67.4)
GAD	147(22.9)
PD	173(27.0)
EPAS, mean (SD)	11.9(4.5)
EQ-5D-5L, mean (SD)	74.4(13.4)

*Entries are n (%) unless indicated differently and present sample characteristics after imputation. GAD, generalized anxiety disorder according to ICD-10 (self-report); GP, general practitioner; PD, panic disorder according to ICD-10 (self-report); EPAS, 6-item subscale Hope and Self-efficacy from the questionnaire for the assessment of Empowerment in Patients with Affective and Schizophrenic disorders; EQ-5D-5L, health state classifier EQ-5D of the EuroQol Group; SD, standard deviation*.

### Treatment preferences for depression in primary care patients

**Figure 1:**
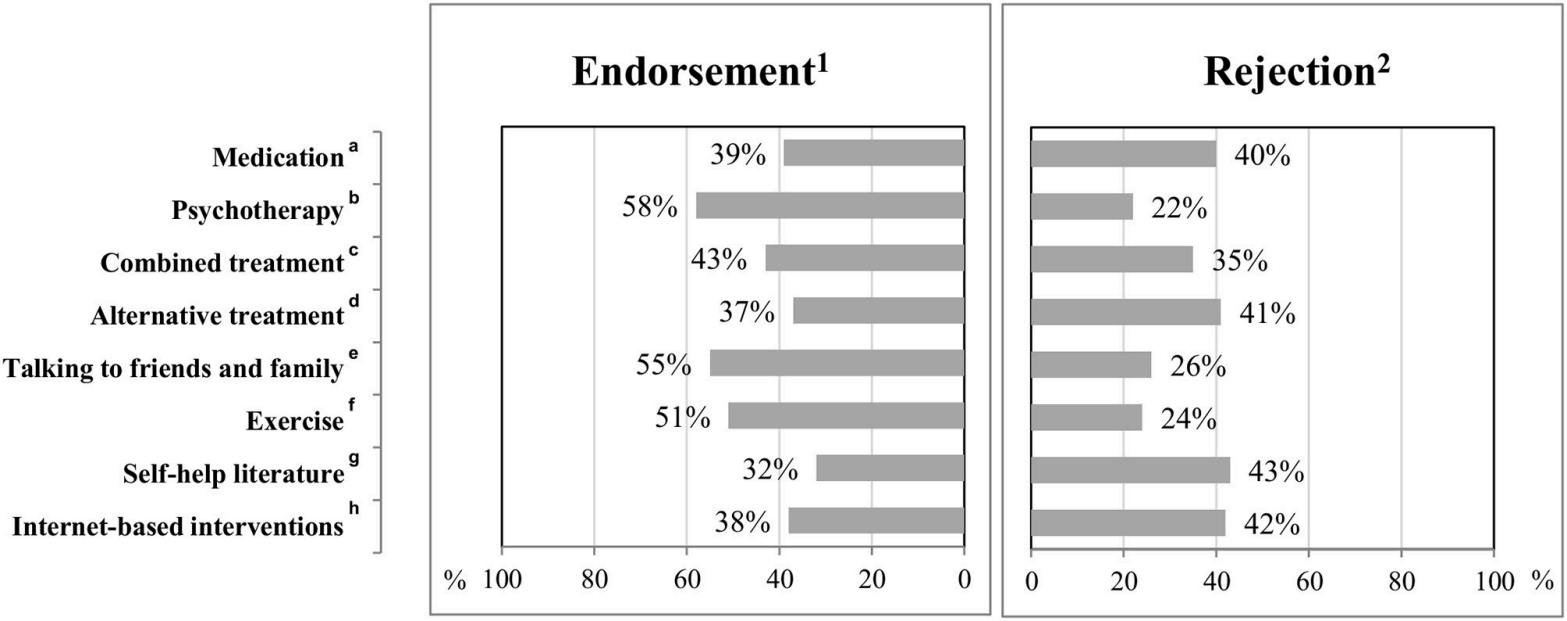
Treatment preferences for depression in primary care. ^1^Endorsement: likely or very likely; ^2^Rejection: unlikely or very unlikely; ^a^*n* = 637; ^b^*n* = 633; ^c^Combined treatment refers to medication and psychotherapy, *n* = 615; ^d^Alternative treatment (e.g. alternative practitioners), *n* = 623; ^e^*n* = 634; ^f^*n* = 638; ^g^*n* = 629; ^h^*n* = 631; percentage of respondents “undecided”: ^a^*n* = 132 (21%); ^b^*n* = 125 (20%); ^c^*n* = 135 (22%); ^d^*n* = 141 (23%); ^e^*n* = 122 (19%); ^f^*n* = 159 (25%); ^g^*n* = 154 (24%); ^h^*n* = 127 (20%).

Figure 1 summarizes ranked treatment preferences for depression. The majority of patients (58%) reported that they were likely or very likely to consider psychotherapy as a treatment option for depression. Similarly, 55 and 51% were likely or very likely to consider talking to friends and family or exercise to manage their depression. Figure S1 in Supplementary Material shows more detailed information about the distributions of patients' treatment preferences.

The mean preference strengths were 3.7 (*SD* = 1.4) for psychotherapy, 3.6 (*SD* = 1.4) for talking to friends and family, 3.5 (*SD* = 1.3) for exercise, 3.1 (*SD* = 1.4) for combined treatment, 3.0 (*SD* = 1.5) for medication, 2.9 for self-help literature (*SD* = 1.4), alternative treatment (*SD* = 1.4) and internet-based interventions (*SD* = 1.5).

### Subgroup differences in treatment preferences for depression concerning age, gender and severity of depression

Table 2 presents results of the subgroup analyses concerning gender-, age-, and depression-related differences in treatment preferences. Gender-related differences were found in the treatment preferences for alternative treatment (*z* = 3.36, *p* = 0.001) and self-help literature (*z* = 3.48, *p* < 0.001) with females showing stronger preferences for these treatment options than men. Regarding age-related differences younger patients preferred psychotherapy to a greater extent than older patients (*z* = −3.71, *p* < 0.001). Patients with moderate depression showed higher preference for medication as a treatment option than patients with mild depression (*z* = −2.68, *p* = 0.007).

**Table 2:** Gender-, age-, and depression-related subgroup differences in treatment preferences for depression in primary care patients.

**Treatment option**	**Gender-related differences**						**Age-related differences**								**Depression-related differences**					
	**fem (*n*)**	**Mean (*SD*)**	**Male (*n*)**	**Mean (*SD*)**	***z***	***p***	**18–30 (*n*)**	**Mean (*SD*)**	**31–50 (*n*)**	**Mean (*SD*)**	**51–82 (*n*)**	**Mean (*SD*)**	***z***	***p***	**Mild (*n*)**	**Mean (*SD*)**	**mod (*n*)**	**Mean (*SD*)**	***z***	***p***
Medication	435	2.98(1.47)	198	3.19(1.44)	−1.68	0.094	137	2.99(1.42)	276	3.01(1.47)	220	3.14(1.47)	1.04	0.298	276	2.87(1.44)	357	3.18(1.46)	−2.68	**0.007**
Psychotherapy	432	3.75(1.34)	197	3.53(1.38)	1.90	0.057	137	3.98(1.21)	276	3.75(1.29)	216	3.39(1.47)	−3.71	<**0.001**	272	3.57(1.37)	357	3.76(1.34)	−1.84	0.065
Combined treatment^a^	416	3.10(1.46)	195	3.14(1.37)	−0.29	0.770	135	3.19(1.42)	264	3.09(1.40)	212	3.08(1.49)	−0.59	0.555	265	3.03(1.42)	346	3.18(1.44)	−1.33	0.184
Alternative treatment^b^	425	3.07(1.43)	195	2.66(1.34)	3.36	**0.001**	136	3.01(1.41)	269	3.11(1.39)	215	2.69(1.42)	−2.46	0.014	270	2.90(1.43)	350	2.97(1.41)	−0.63	0.528
Talking to friends and family	432	3.64(1.39)	198	3.46(1.45)	1.43	0.152	138	3.57(1.42)	274	3.65(1.38)	218	3.51(1.45)	−0.39	0.698	273	3.62(1.35)	357	3.56(1.46)	0.19	0.852
Exercise	435	3.53(1.31)	199	3.48(1.36)	0.42	0.675	138	3.61(1.28)	277	3.56(1.29)	219	3.40(1.40)	−1.42	0.155	275	3.51(1.36)	359	3.52(1.30)	−0.04	0.965
Self-help literature	430	3.01(1.40)	196	2.59(1.24)	3.48	<**0.001**	137	2.68(1.31)	274	2.88(1.34)	215	3.00(1.43)	2.05	0.040	274	2.88(1.33)	352	2.88(1.40)	0.14	0.887
Internet-based interventions	431	2.89(1.50)	197	2.86(1.39)	0.18	0.860	137	3.16(1.37)	278	3.02(1.45)	213	2.52(1.48)	−4.30	<**0.001**	275	2.74(1.43)	353	2.99(1.49)	−2.13	0.033

a *Combined treatment refers to medication and psychotherapy*.

b *Alternative treatment (e.g., alternative practitioners); SD, standard deviation; treatment preferences had a possible range from 1 to 5, with higher scores indicating stronger preference; gender- and depression-related differences were analyzed with Wilcoxon two-sample tests; age-related differences were calculated using Cuzicks Trend test; the level of significance was p < 0.01*.

### Associated variables of treatment preferences for depression

Whereas several sociodemographic and illness-related variables were associated with treatment preferences for depression, work-related variables, i.e., the patients' vocational qualification did not show significant impact on any treatment option (Table 3).

**Table 3:** Associated variables of treatment preferences for depression in primary care patients.

	**Medication**				**Psychotherapy**				**Combined treatment^a^**				**Alternative treatment^b^**			
**Sociodemographic and work-related variables**	**OR (95% CI)**	**p**	**Wald-Chi**^2^	**p**> **Chi**^2^	**OR (95% CI)**	**p**	**Wald-Chi**^2^	**p**> **Chi**^2^	**OR (95% CI)**	**p**	**Wald-Chi**^2^	**p**> **Chi**^2^	**OR (95% CI)**	**p**	**Wald-Chi**^2^	**p**> **Chi**^2^
male	1.45 (1.06, 1.98)	0.019			0.80 (0.58, 1.10)	0.167			1.12 (0.82, 1.53)	0.476			0.63 (0.46, 0.87)	**0.005**		
age	1.01 (1.01, 1.02)	0.033			1.00 (0.99, 1.01)	0.726			1.01 (1.00, 1.02)	0.027			1.00 (0.99, 1.00)	0.229		
marital status (R: married)			1.66	0.436			11.14	**0.004**			7.17	0.028			1.33	0.513
single	0.98 (0.70, 1.38)	0.907			1.82 (1.28, 2.58)	**0.001**			1.32 (0.94,1.86)	0.110			1.14 (0.81, 1.61)	0.447		
divorced/widowed	0.77 (0.51, 1.16)	0.212			1.17 (0.76, 1.80)	0.486			0.70 (0.45, 1.09)	0.117			1.26 (0.82, 1.93)	0.286		
education (R: low)			0.17	0.920			11.08	**0.004**			3.70	0.157			3.39	0.183
middle	1.09 (0.68, 1.72)	0.730			2.04 (1.29, 3.23)	**0.002**			1.52 (0.96, 2.40)	0.072			1.47 (0.93, 2.31)	0.099		
high	1.11 (0.65, 1.91)	0.696			2.27 (1.32, 3.88)	**0.003**			1.58 (0.93, 2.68)	0.093			1.59 (0.93, 2.70)	0.091		
Vocational qualification (R: none)			2.63	0.621			1.33	0.857			1.44	0.837			0.12	0.998
still undergoing training	0.93 (0.36, 2.38)	0.875			1.65 (0.62, 4.38)	0.318			1.06 (0.42, 2.70)	0.900			0.87 (0.34, 2.22)	0.774		
completed apprenticeship	0.98 (0.51, 1.88)	0.941			1.29 (0.69, 2.40)	0.425			1.14 (0.61, 2.15)	0.681			0.92 (0.48, 1.74)	0.792		
secondary vocational education	1.00 (0.48, 2.08)	0.996			1.18 (0.58, 2.41)	0.654			1.14 (0.55, 2.33)	0.729			0.94 (0.46, 1.95)	0.877		
university degree	0.67 (0.31, 1.44)	0.302			1.17 (0.56,2.48)	0.673			0.85 (0.40, 1.80)	0.672			0.95 (0.44, 2.02)	0.885		
**Illness-related variables**																
Moderate depression (R: mild)	1.25 (0.92, 1.68)	0.150			1.24 (0.91, 1.69)	0.179			1.05 (0.78, 1.43)	0.734			1.08 (0.79, 1.46)	0.636		
Treatment history	0.51 (0.38, 0.70)	<**0.001**			0.74 (0.54, 1.02)	0.064			0.62 (0.45, 0.84)	**0.002**			1.27 (0.93, 1.74)	0.132		
GAD	1.11 (0.79, 1.55)	0.548			1.30 (0.91, 1.85)	0.145			1.22 (0.87, 1.71)	0.250			1.65 (1.17, 2.34)	**0.005**		
PD	1.09 (0.78, 1.51)	0.628			1.32 (0.93, 1.87)	0.126			1.21 (0.87, 1.69)	0.265			1.27 (0.90, 1.79)	0.167		
EPAS	0.94 (0.91, 0.97)	<**0.001**			0.98 (0.95, 1.01)	0.177			0.95 (0.92, 0.98)	**0.002**			1.01 (0.98, 1.04)	0.493		
EQ-5D-5L	0.99 (0.98, 1.00)	0.081			1.00 (0.99, 1.01)	0.832			0.99 (0.98, 1.00)	0.102			1.01 (1.00, 1.02)	0.140		
N	633				629				611				620			
Pseudo R^2^	0.03				0.03				0.03				0.02			
	**Talking to friends and family**				**Exercise**				**Self-help literature**				**Internet-based self-help programs**			
**Sociodemographic and work-related variables**	OR (95% CI)	P	Wald-Chi^2^	p> Chi^2^	OR (95% CI)	P	Wald-Chi^2^	p> Chi^2^	OR (95% CI)	p	Wald-Chi^2^	p> Chi^2^	OR (95% CI)	P	Wald-Chi^2^	p> Chi^2^
male	0.88 (0.64, 1.21)	0.436			0.99 (0.72, 1.36)	0.955			0.66 (0.48, 0.90)	**0.009**			1.13 (0.83, 1.55)	0.444		
age	1.00 (0.99, 1.01)	0.670			1.00 (0.99, 1.01)	0.834			1.00 (0.99, 1.01)	0.863			0.99 (0.98, 0.99)	**0.003**		
marital status (R: married)			2.61	0.272			1.32	0.516			3.63	0.163			0.13	0.937
single	1.33 (0.94, 1.88)	0.108			1.11 (0.79, 1.56)	0.553			1.35 (0.96, 1.90)	0.082			1.03 (0.73, 1.45)	0.882		
divorced/widowed	1.08 (0.70, 1.66)	0.731			0.85 (0.55, 1.31)	0.460			1.32 (0.87, 2.01)	0.195			1.08 (0.70, 1.68)	0.720		
education (R: low)			5.79	0.055			30.11	<**0.001**			9.68	**0.008**			16.48	<**0.001**
middle	1.69 (1.06, 2.71)	0.029			2.19 (1.37, 3.49)	**0.001**			2.06 (1.30, 3.25)	**0.002**			1.85 (1.16, 2.96)	0.010		
high	1.84 (1.06, 3.19)	0.029			4.77 (2.73, 8.35)	<**0.001**			1.69 (0.98, 2.89)	0.058			3.10 (1.79, 5.34)	<**0.001**		
Vocational qualification (R: none)			0.86	0.930			1.43	0.838			5.30	0.257			7.89	0.096
still undergoing training	1.19 (0.45, 3.19)	0.723			0.56 (0.21, 1.53)	0.260			1.70 (0.64, 4.49)	0.283			3.10 (1.21, 7.94)	0.018		
completed apprenticeship	1.33 (0.69, 2.55)	0.391			0.73 (0.37, 1.44)	0.366			2.13 (1.07, 4.23)	0.031			1.50 (0.78, 2.89)	0.225		
secondary vocational education	1.32 (0.63, 2.77)	0.464			0.70 (0.33, 1.50)	0.359			2.06 (0.96, 4.41)	0.063			1.41 (0.67, 2.94)	0.362		
university degree	1.19 (0.55, 2.56)	0.663			0.75 (0.34, 1.66)	0.475			2.35 (1.05, 5.25)	0.037			1.07 (0.49, 2.32)	0.862		
**Illness-related variables**																
Moderate depression (R: mild)	1.21 (0.88, 1.64)	0.237			1.21 (0.89, 1.66)	0.219			1.03 (0.76, 1.39)	0.839			1.20 (0.89, 1.63)	0.232		
Treatment history	0.93 (0.67, 1.27)	0.632			1.08 (0.79, 1.49)	0.619			1.04 (0.77, 1.42)	0.786			0.96 (0.71, 1.31)	0.818		
GAD	1.11 (0.77, 1.61)	0.585			1.53 (1.07, 2.18)	0.019			1.20 (0.85, 1.68)	0.302			2.06 (1.47, 2.91)	<**0.001**		
PD	1.10 (0.77, 1.56)	0.603			1.34 (0.95, 1.90)	0.095			1.00 (0.71, 1.39)	0.977			1.41 (1.00, 2.00)	0.053		
EPAS	1.10 (1.06, 1.14)	<**0.001**			1.12 (1.08, 1.16)	<**0.001**			1.04 (1.00, 1.07)	0.025			1.01 (0.98, 1.05)	0.458		
EQ-5D-5L	1.00 (0.99, 1.01)	0.742			1.01 (1.00, 1.02)	0.253			1.00 (0.99, 1.01)	0.945			1.00 (0.99, 1.01)	0.434		
N	630				634				626				628			
Pseudo R^2^	0.02				0.05				0.02				0.04			

a *Combined treatment refers to medication and psychotherapy*.

b *Alternative treatment (e.g., alternative practitioners); CI, confidence interval; OR, odds ratio; R, reference category; Wald-Chi^2^, Wald Chi^2^ test for testing the joint significance of categorical indicators; the level of significance was p < 0.01*.

#### Sociodemographic variables associated with treatment preferences for depression

The patients' educational level was significantly associated with treatment preferences for psychotherapy, exercise and self-help literature. Hence, patients with a higher academic education were more likely to prefer these treatment options. For example, patients with a high educational level had 2.27 (95%-CI = 1.32, 3.88) higher odds for preferring psychotherapy than patients with a low educational level, given that all other variables in the model are held constant. Gender was associated with alternative treatment and self-help literature. Thus, women had 1/0.63 = 1.59 higher odds (95%-CI = 1.15, 2.17) for expressing stronger preference toward alternative treatment and 1/0.66 = 1.52 higher odds (95%-CI = 1.11, 2.08) for preferring self-help literature. The patients' marital status emerged as a factor that was significantly associated with a higher preference for psychotherapy as singles were more likely to favor psychotherapy than married patients (OR = 1.82; 95%-CI = 1.28, 2.58).

#### Illness-related variables associated with treatment preferences for depression

Patients scoring higher on the EPAS hope and self-efficacy subscale were less likely to prefer medication (OR = 0.94; 95%-CI = 0.91, 0.97) or combined treatment (OR = 0.95; 95%-CI = 0.92, 0.98), but had higher odds for exercise (OR = 1.12; 95%-CI = 1.08, 1.16) or talking to friends and family (OR = 1.10; 95%-CI = 1.06, 1.14). Having a comorbid general anxiety disorder increased the willingness to seek alternative treatment (OR = 1.65; 95%-CI = 1.17, 2.34) and internet-based interventions (OR = 2.06; 95%-CI = 1.47, 2.91). Finally, patients who had not received treatment due to emotional stress in the past were more likely to score high on medication (OR = 1.96; 95%-CI = 1.43, 2.63) or combined treatment (OR = 1.61; 95%-CI = 1.19, 2.22) than patients who did receive help due to psychological problems before. The following illness-related factors were not significantly associated with any treatment preference: severity of depression, comorbid panic disorder and HRQOL.

### Internet-based interventions as a treatment option for depression

About 38% of the patients were likely or very likely to consider internet-based interventions in case of depression. In contrast, 42% of the patients were unlikely or very unlikely to prefer internet-based interventions for the treatment of depression. Subgroup differences were found for age, as younger patients expressed a stronger treatment preference for internet-based interventions than older patients (*z* = −4.30, *p* < 0.001). Patients with moderate depression were more likely to prefer internet-based interventions than patients with mild depression (*z* = −2.13, *p* = 0.033), even though this was not significant given the significance level of *p* < 0.01. Associated factors for internet-based interventions were the patients' age, educational level and having a comorbid anxiety disorder. Thus, younger patients were significantly more likely to express stronger acceptance toward internet-based interventions than older patients (OR = 1.01; 95%-CI = 1.01, 1.02). In addition, patients with a high educational level had 3.10 (95%-CI = 1.79, 5.34) higher odds of scoring higher on internet-based interventions than patients with a low educational level. Finally, those with a comorbid general anxiety disorder had higher preferences for internet-based interventions (OR = 2.06; 95%-CI = 1.47, 2.91).

## Discussion

### Treatment preferences for depression in primary care patients

In the present study, the strongest treatment preferences were reported for psychotherapy, talking to friends and family, and exercise. In this regard, patients expressed stronger preference for psychotherapy than for medication or combined treatment (psychotherapy and medication). Our observed mean values for psychotherapy and medication are in line with Raue et al. (26) who found a mean preference strength of 4.1 for psychotherapy and 2.9 for antidepressants using 5-point Likert scales. A large number of previous studies showed that patients often prefer psychotherapy to medication for the treatment of depression (7, 27–34). Despite the known effectiveness of antidepressants (35), low acceptance rates were reported in previous studies (36, 37) which may be explained by general reluctance or anticipated side effects caused by the drugs.

Our finding for highly favorable preferences toward other treatment options such as talking to friends and family is supported by previous research reporting that individuals prefer informal help from a confidant to formal sources of help (17, 38, 39). Existing literature indicates that positively experienced lay support may effectively help to overcome symptoms of depression (40, 41). In a qualitative study (42), *n* = 417 participants who had sought help for depression from family or friends filled in a questionnaire about advantages and disadvantages of informal support. The most frequently reported benefit was social support with emotional support being the most commonly cited type of support, followed by informational, companionship and instrumental support. On the other hand, the most salient barriers in seeking help from a confidant were seen in issues of stigma, such as stigmatizing responses or anticipated stigma, as well as inappropriate support and lack of knowledge, training and expertise.

Regarding physical exercise, a Cochrane review (43) including 35 trials found a moderate clinical effect, when comparing exercise to no treatment or a control intervention [pooled standardized mean difference (SMD) = −0.62]. Trials of high methodological quality indicated smaller effects, but there is ongoing research in this field providing growing evidence for the effectiveness of exercise in the treatment of depression. For example, a large effectiveness trial conducted in mild to moderately depressed primary care patients indicates that adjunctive physical exercise is more effective than treatment in primary healthcare alone (44).

### Subgroup differences in treatment preferences for depression concerning age, gender and severity of depression

We found no gender-related differences regarding treatment preferences for psychotherapy which is in contrast with many previous studies indicating that women are more likely than men to favor psychotherapy (7, 29, 30, 34, 45, 46). Houle et al. (34) point out that a possible explanation for this preference may be that women express and talk about their feelings more easily. In terms of gender differences, women in our study showed stronger preferences for alternative treatment and self-help literature which is in line with other studies (47, 48).

Our finding that younger age groups were more likely than older patients to prefer psychotherapy is supported by Boehlen et al. (49) showing that the willingness to seek help for psychological problems was lower in older age groups compared to younger study participants. Barriers to see a psychotherapist for depression reported by elderly individuals were the wish to solve the emotional problems autonomously and fear of stigma (50).

Regarding severity of depression, we found that more severe depression was associated with stronger preferences for the intake of medication. This may be due to higher psychological strain in stronger affected patients. In line with this finding, Berner et al. (36) found that increasing symptom severity was associated with stronger treatment preferences for interventions that had to be initiated by a health care professional. On the other hand, the authors note that a large number of both affected and unaffected individuals do not understand depression as a treatable disorder. Even in patients who are strongly affected by depressive symptoms, almost half of them did not wish to be treated.

### Associated variables of treatment preferences for depression

The patients' educational level was the most salient sociodemographic factor associated with treatment preferences for depression. Accordingly, a higher educational level was associated with stronger treatment preferences. Likewise, patients with higher education have been found to use face-to-face psychotherapy more frequently than patients with lower education (51). This may be explained by increased health literacy in patients with more formal education (52–54). Knowledge about the effectiveness of therapy options or treatment components for mental illnesses may influence patients' attitudes and result in stronger willingness to consider these options. In accordance with a previous study (34) we found that higher education was significantly associated with preference for psychotherapy but not for medication. As a possible explanation, Houle et al. (34) point out that, unlike taking antidepressants, attending psychotherapy requires a high degree of self-reflection and patience to overcome symptoms of depression, attributes which may be more pronounced in people with a higher educational level.

In accordance with subgroup analyses within the present study, female gender was associated with increased preferences for alternative therapy and self-help literature. Another sociodemographic factor influencing treatment preference was the patients' marital status. The finding that singles were more likely to express more preferences for psychotherapy than married patients may be explained by lacking emotional support from the spouse in patients without a partner, leading to a stronger wish to talk to a psychotherapist and receive professional support (55). Furthermore, Kessler et al. (56) found out that singles who had never been married before, had a higher likelihood of seeking support from a mental health specialist.

Concerning illness-related factors for treatment preferences of depression, the patients' self-efficacy, signifying the patients' beliefs to be able to change something about their situation, seems to be of particular importance. Hence, higher self-efficacy was associated with less favorable preferences toward medication and combined treatment, which involve a passive component of dealing with the disease, but with higher preferences for active treatment options, i.e., doing sports and talking to friends and family. These findings may be associated with patient beliefs about depression etiology (12). Accordingly, previous research indicates that individuals who favor medication to psychotherapy tend to attribute their depression more to biomedical causes (12, 29, 46) and were less likely to consider pessimistic thinking as a cause for their depression (57). Therefore, patients who show high preferences for medication and low preferences for exercise or talking to a confidant may underestimate the potential of self-efficacy.

Moreover, having a treatment history of depression was associated with reduced preference for medication or combined treatment. This may be caused by negative experiences with previous intake of medication as patients with positive experience of a certain treatment method are more likely to seek the same treatment in the future (1).

### Internet-based interventions as a treatment option for depression

During the last decade e-mental health interventions have been a rapidly developing field of research. Christensen et al. (58) define e-mental health as “mental health services and information delivered or enhanced through the Internet and related technologies” (p. 17). In this regard, iCBT represents a new, innovative and effective treatment approach for mental health disorders. Systematic reviews, meta-reviews and meta-analyses point out the effectiveness and user acceptance of iCBT programs for depression (59–63). E-mental health interventions such as iCBT programs may therefore function as a clinically effective and cost-effective add-on treatment component besides the existing somato-, psycho- and psychosocial-therapy treatments within stepped care of depression (64, 65). In Germany, however, these new approaches have hardly been implemented into the German health care system due to legal barriers (66). In addition, freely available, low threshold e-mental health interventions for the adjunctive treatment of depression are still at an early stage of dissemination.

Within the present study, more than a third of the patients were likely or very likely to consider internet-based interventions in case of depression. In contrast, a slightly higher number of patients reported the opposite. Hence, internet-based interventions seem to evoke opposing reactions in primary care patients with mild to moderate depression. Nevertheless, these findings indicate that there is a considerable amount of patients showing interest in using e-mental health interventions for the treatment of depression. A previous study (67) investigating the implementation of the internet-based self-management program moodgym in inpatient psychiatric clinics found that stronger treatment preference for internet-based interventions was a significant predictor for starting the moodgym program. In view of influential sociodemographic factors, age and education were associated with preferences for internet-based interventions. Findings regarding the patients' age are in line with results from Batterham and Calear (68), who found that younger patients were more likely to prefer internet-based interventions ahead of face-to-face-therapy compared to older patients. This is most likely due to the fact that the amount of younger individuals using the internet for private purposes is higher compared to older individuals (69) and younger people use the internet more frequently than do older age groups (70). Likewise, Eichenberg et al. (71) found out that age as well as internet usage corresponded with people's willingness to use e-mental health services. Hence, older patients may be less familiar with the internet in general and may feel that they do not have sufficient computer skills to conduct an online program. In a qualitative study investigating patients with obesity and comorbid depression, Löbner et al. (72) found that according to clinical experts, the patients' age was cited as an important characteristic that needs to be considered when implementing internet-based interventions in rehabilitative care. Furthermore, Batterham and Calear (68) suggest that older individuals and also those with a lower educational level should be made more familiar with internet-based interventions. The association between higher education and stronger preferences for internet-based programs is supported by previous research (68, 71, 73, 74). People with increased education may be more aware of treatment options for depression in general, which may contribute to more positive attitudes toward internet-based treatment. Additionally, compared to patients with a high educational level, those with lower education have been found to be more likely to drop out from internet-based CBT interventions (75) or trials investigating the effectiveness and acceptability of iCBT (76).

As an illness-related factor, comorbid general anxiety disorder was found to be associated with stronger treatment preferences for internet-based interventions. A possible reason may be increased psychological stress in patients with comorbid anxiety resulting in greater perceived need for help (77). Another explanation may be that patients suffering from anxiety disorders may endorse the anonymity of online programs (78). In this regard, individuals reported that providing anonymous programs may have the effect that more people dare to seek help and that conducting an online program may be less embarrassing than face-to-face therapy (79). Klein and Cook (80) found out that individuals preferring online interventions had more stigmatizing beliefs, lower scores on extraversion and emotional stability, characteristics which may be more common in patients with comorbid anxiety.

### Strengths and limitations

Strengths of this study are the large sample, the naturalistic setting in primary care and the extended assessment of treatment preferences for depression including analyses on new media based approaches. However, this study also has some limitations. First, since we recruited primary care patients with mild or moderate depression, our findings may not apply to patients with severe depression and cannot be generalized to settings other than primary care. Treatment preferences reported by primary care patients may differ from those seen in specialized care settings. Moreover, we cannot ensure generalizability of our results for the whole population of primary care patients since the proportion of older patients in our study is not representative for this population. However, we were able to recruit a sufficient number of individuals in old age and we had enough power to obtain reliable results in this age range. Second, data were collected between 2014 and 2015, when internet-based interventions were hardly disseminated in public mental health care in Germany. Despite slow implementation, patients' perspectives on internet-based interventions might have changed since that time due to increasing public relations work. Third, patients were asked to indicate their treatment preferences for internet-based interventions in case of depression. However, the term internet-based interventions comprises many different aspects and may refer to guided or unguided self-management programs, mobile self-help apps, e-mail therapy, videoconference-based counseling or chat groups. In this regard, a scoping review (81) investigating public acceptability and service preferences of e-mental health services in four studies (71, 80, 82, 83) showed that most people from the general population preferred guided over unguided programs. In extension of this research, future research should investigate treatment preferences for e-mental health interventions with regard to different application types (e.g., internet- vs. mobile-based), guidance (guided vs. self-guided), costs (free availability vs. self-payment or reimbursement models) and form of communication (synchronous vs. asynchronous) in clinical populations, e.g., patients with depressive symptoms. Third, the included sociodemographic, illness-related and work-related factors yielded a low prediction of variance. Hence, we possibly missed to gather information about other variables of potential influence. These may include perceived stigma (80, 84, 85), perceived barriers to receive treatment (86) such as living in rural communities (50), vicarious experience with depression (1) and beliefs about etiology of depression (12). Future studies should take these factors into account to gain more knowledge about factors attributing to depression treatment preferences. Moreover, future studies should intensify research on reasons leading to non-preference of e-mental health interventions in primary care patients in order to address potential barriers.

## Conclusions

To our knowledge, this is the first study investigating preferences for a broad range of treatment options including internet-based interventions for depression in primary care patients. Our results underline the importance of active patient involvement in order to find the perfect match between individual patient preferences and existing treatment options for depression. Since the patients' education and self-efficacy seemed to influence preferences for a variety of different treatment options, these factors may be particularly considered by GPs within the process of shared decision-making. In this regard, GPs may point out the positive effects of self-efficacy and empowerment on treatment success and recovery to their patients. To increase the patients' health literacy, patients should be informed thoroughly about the effectiveness and clinical evidence of treatment options for depression, e.g., with the help of information brochures. These should include information about internet-based interventions as patients may only have little knowledge about these new approaches.

Future research may investigate how treatment preferences for innovative treatment options such as internet-based self-management programs affect the adherence to and effectiveness of interventions based on new media.

## Data availability

The raw data supporting the conclusions of this manuscript will be made available by the authors upon request.

## Author contributions

MD, ML, and SR-H: conceptualized the paper; AP: conducted the statistical analyses; MD: wrote the paper; ML, JS, AP, and SR-H: revised it critically for important intellectual content.

### Conflict of interest statement

The authors declare that the research was conducted in the absence of any commercial or financial relationships that could be construed as a potential conflict of interest.

## References

[1] Berkowitz SA Bell RA Kravitz RL Feldman MD. Vicarious experience affects patients' treatment preferences for depression. PLoS ONE (2012) 7:e31269. 10.1371/journal.pone.0031269 22363603 PMC3283627

[2] Gensichen J Huchzermeier C Aldenhoff JB Gerlach FM Hinze-Selch D. Signals for the initiation of structured diagnostic procedures for depression in primary health care. A practice-relevant evaluation of international guidelines. Z Ärztl Fortbild Qualitätssich. (2005) 99:57–63. 15804131

[3] Jacobi F Höfler M Meister W Wittchen HU. Prevalence, detection and prescribing behavior in depressive syndromes. A German federal family physician study. Nervenarzt (2002) 73:651–8. 10.1007/s00115-002-1299-y 12212528

[4] Goldman LS Nielsen NH Champion HC. Awareness, diagnosis, and treatment of depression. J Gen Intern Med. (1999) 14:569–80. 10.1046/j.1525-1497.1999.03478.x 10491249 PMC1496741

[5] Linden M Maier W Achberger M Herr R Helmchen H Benkert O. Psychiatric diseases and their treatment in general practice in Germany. Results of a World Health Organization (WHO) study. Nervenarzt (1996) 67:205–15. 8901278

[6] Pincus HA Davis WW McQueen LE. ‘Subthreshold’ mental disorders. Br J Psychiatry (1999) 174:288–96. 10533546 10.1192/bjp.174.4.288

[7] Churchill R Khaira M Gretton V Chilvers C Dewey M Duggan C. Treating depression in general practice: factors affecting patients' treatment preferences. Br J Gen Pract. (2000) 50:905–6. 11141877 PMC1313855

[8] Gelhorn HL Sexton CC Classi PM. Patient preferences for treatment of major depressive disorder and the impact on health outcomes: a systematic review. Prim Care Companion CNS Disord. (2011) 13:PCC.11r01161. 10.4088/PCC.11r01161 22295273 PMC3267514

[9] DGPPN, BÄK, KBV, AWMF for the guideline group unipolar depression S3-Leitlinie/Nationale VersorgungsLeitlinie Unipolare Depression – Langfassung, 2. Auflage. Version 5 (2015). Available online at: http://www.leitlinien.de/mdb/downloads/nvl/depression/depression-2aufl-vers5-lang.pdf (Accessed Augest 10, 2017).

[10] Härter M Loh A Spies C Gemeinsam entscheiden - erfolgreich behandeln. Neue Wege für Ärzte und Patienten im Gesundheitswesen. Köln: Deutscher Ärzte-Verlag (2005).

[11] Elwyn G Edwards A Mowle S Wensing M Wilkinson C Kinnersley P. Measuring the involvement of patients in shared decision-making: a systematic review of instruments. Patient Educ Couns. (2001) 43:5–22. 10.1016/S0738-3991(00)00149-X 11311834

[12] Steidtmann D Manber R Arnow BA Klein DN Markowitz JC Rothbaum BO. Patient treatment preference as a predictor of response and attrition in treatment for chronic depression. Depress Anxiety (2012) 29:896–905. 10.1002/da.21977 22767424 PMC3463778

[13] Johansson R Nyblom A Carlbring P Cuijpers P Andersson G. Choosing between Internet-based psychodynamic versus cognitive behavioral therapy for depression: a pilot preference study. BMC Psychiatry (2013) 13:268. 10.1186/1471-244X-13-268 24139066 PMC3852703

[14] Schuster R Berger T Laireiter A-R Computer und Psychotherapie – geht das zusammen? Psychotherapeut (2017) 75:e695 10.1007/s00278-017-0214-8.

[15] Kleiboer A Smit J Bosmans J Ruwaard J Andersson G Topooco N. European COMPARative Effectiveness research on blended Depression treatment versus treatment-as-usual (E-COMPARED): study protocol for a randomized controlled, non-inferiority trial in eight European countries. Trials (2016) 17:387. 10.1186/s13063-016-1511-1 27488181 PMC4972947

[16] Luck-Sikorski C Stein J Heilmann K Maier W Kaduszkiewicz H Scherer M. Treatment preferences for depression in the elderly. Int Psychogeriatr. (2017) 29:389–98. 10.1017/S1041610216001885 27890036

[17] Angermeyer MC Matschinger H Riedel-Heller SG. Whom to ask for help in case of a mental disorder? Preferences of the lay public. Soc Psychiatry Psychiatr Epidemiol. (1999) 34:202–10. 10.1007/s001270050134 10365626

[18] Brauns H Steinmann S Educational reform in France, West-Germany and the United Kingdom: updating the CASMIN educational classification. ZUMA Nachrichten (1999) 23:7–44.

[19] Löwe B Spitzer R Zipfel S Herzog W Gesundheitsfragebogen für Patienten (PHQ D). Komplettversion und Kurzform. Testmappe mit Manual, Fragebögen, Schablonen. 2. Auflage: Pfizer (2002).

[20] Spitzer RL Williams JB Kroenke K Linzer M deGruy FV3 Hahn SR. Utility of a new procedure for diagnosing mental disorders in primary care. The PRIME-MD 1000 study. JAMA (1994) 272:1749–56. 10.1001/jama.1994.03520220043029 7966923

[21] Kilian R Becker T Schleuning G Welschehold M Hertle C Matschinger H Die Entwicklung eines Standardisierten Verfahrens zur Messung von Empowerment im Prozess der psychiatrischen Behandlung von Patienten mit Schweren Psychischen Erkrankungen. Ulm: Abschlussbericht Förderkennz 101GX0743 (2012).

[22] Herdman M Gudex C Lloyd A Janssen M Kind P Parkin D. Development and preliminary testing of the new five-level version of EQ-5D (EQ-5D-5L). Qual Life Res. (2011) 20:1727–36. 10.1007/s11136-011-9903-x 21479777 PMC3220807

[23] White IR Royston P Wood AM. Multiple imputation using chained equations: issues and guidance for practice. Stat Med. (2011) 30:377–99. 10.1002/sim.4067 21225900

[24] Cuzick J. A Wilcoxon-type test for trend. Stat Med. (1985) 4:87–90. 10.1002/sim.4780040112 3992076

[25] Wolfe R Gould W An approximate likelihood-ratio test for ordinal response models. Stata Tech Bull. (1998) 7:1–52.

[26] Raue PJ Schulberg HC Heo M Klimstra S Bruce ML. Patients' depression treatment preferences and initiation, adherence, and outcome: a randomized primary care study. Psychiatr Serv. (2009) 60:337–43. 10.1176/ps.2009.60.3.337 19252046 PMC2710876

[27] Van Schaik DJF Klijn AFJ van Hout HPJ van Marwijk HWJ Beekman ATF Haan M de. Patients' preferences in the treatment of depressive disorder in primary care. Gen Hosp Psychiatry (2004) 26:184–9. 10.1016/j.genhosppsych.2003.12.001 15121346

[28] Löwe B Schulz U Gräfe K Wilke S. Medical patients' attitudes toward emotional problems and their treatment. What do they really want? J Gen Intern Med. (2006) 21:39–45. 10.1111/j.1525-1497.2005.0266.x 16423121 PMC1484618

[29] Khalsa S-R McCarthy KS Sharpless BA Barrett MS Barber JP. Beliefs about the causes of depression and treatment preferences. J Clin Psychol. (2011) 67:539–49. 10.1002/jclp.20785 21365652

[30] Dwight-Johnson M Sherbourne CD Liao D Wells KB. Treatment preferences among depressed primary care patients. J Gen Intern Med. (2000) 15:527–34. 10.1046/j.1525-1497.2000.08035.x 10940143 PMC1495573

[31] Iacoviello BM McCarthy KS Barrett MS Rynn M Gallop R Barber JP. Treatment preferences affect the therapeutic alliance: implications for randomized controlled trials. J Consult Clin Psychol. (2007) 75:194–8. 10.1037/0022-006X.75.1.194 17295580

[32] Mergl R Henkel V Allgaier A-K Kramer D Hautzinger M Kohnen R. Are treatment preferences relevant in response to serotonergic antidepressants and cognitive-behavioral therapy in depressed primary care patients? Results from a randomized controlled trial including a patients' choice arm. Psychother Psychosom. (2011) 80:39–47. 10.1159/000318772 20975325

[33] Wittink MN Cary M Tenhave T Baron J Gallo JJ. Towards patient-centered care for depression: conjoint methods to tailor treatment based on preferences. Patient (2010) 3:145–57. 10.2165/11530660-000000000-00000 20671803 PMC2910930

[34] Houle J Villaggi B Beaulieu M-D Lespérance F Rondeau G Lambert J. Treatment preferences in patients with first episode depression. J Affect Disord. (2013) 147:94–100. 10.1016/j.jad.2012.10.016 23167975

[35] Cipriani A Furukawa TA Salanti G Chaimani A Atkinson LZ Ogawa Y. Comparative efficacy and acceptability of 21 antidepressant drugs for the acute treatment of adults with major depressive disorder: A systematic review and network meta-analysis. Lancet (2018) 391:1357–66. 10.1016/S0140-6736(17)32802-7 29477251 PMC5889788

[36] Berner MM Kriston L Sitta P Härter M. Treatment of depressive symptoms and attitudes towards treatment options in a representative German general population sample. Int J Psychiatry Clin Pract. (2008) 12:5–10. 10.1080/13651500701330783 24916490

[37] Hegerl U Althaus D Stefanek J. Public attitudes towards treatment of depression: effects of an information campaign. Pharmacopsychiatry (2003) 36:288–91. 10.1055/s-2003-45115 14663652

[38] Highet NJ Hickie IB Davenport TA. Monitoring awareness of and attitudes to depression in Australia. Med J Aust. (2002) 176(Suppl. 8):63–8. 12065000 10.5694/j.1326-5377.2002.tb04506.x

[39] Gabriel A Violata C Depression literacy among patients and the public: a literature review. Primary Psychiatry (2010) 17:55–64.

[40] Zuroff DC Blatt SJ Vicissitudes of life after the short-term treatment of depression: roles of stress, social support, and personality. J Soc Clin Psychol. (2002) 21:473–96. 10.1521/jscp.21.5.473.22622

[41] Nasser EH Overholser JC. Recovery from major depression: the role of support from family, friends, and spiritual beliefs. Acta Psychiatr Scand. (2005) 111:125–32. 10.1111/j.1600-0447.2004.00423.x 15667431

[42] Griffiths KM Crisp DA Barney L Reid R. Seeking help for depression from family and friends: a qualitative analysis of perceived advantages and disadvantages. BMC Psychiatry (2011) 11:196. 10.1186/1471-244X-11-196 22171567 PMC3271042

[43] Cooney GM Dwan K Greig CA Lawlor DA Rimer J Waugh FR Exercise for depression. Cochrane Database Syst Rev. (2013) 9:1–160. 10.1002/14651858 PMC9721454 24026850

[44] Hallgren M Kraepelien M Öjehagen A Lindefors N Zeebari Z Kaldo V. Physical exercise and internet-based cognitive-behavioural therapy in the treatment of depression: Randomised controlled trial. Br J Psychiatry (2015) 207:227–34. 10.1192/bjp.bp.114.160101 26089305

[45] Givens JL Houston TK van Voorhees BW Ford DE Cooper LA. Ethnicity and preferences for depression treatment. Gen Hosp Psychiatry (2007) 29:182–91. 10.1016/j.genhosppsych.2006.11.002 17484934

[46] Fernandez y Garcia E Franks P Jerant A Bell RA Kravitz RL. Depression treatment preferences of Hispanic individuals: exploring the influence of ethnicity, language, and explanatory models. J Am Board Fam Med. (2011) 24:39–50. 10.3122/jabfm.2011.01.100118 21209343 PMC3061814

[47] Rhee TG Harris IM. Gender differences in the use of complementary and alternative medicine and their association with moderate mental distress in U.S. adults with migraines/severe headaches. Headache (2017) 57:97–108. 10.1111/head.12986 27885674

[48] McLean S Kapell B She reads, he reads: gender differences and learning through self-help books. RELA (2015) 6:55–72. 10.3384/rela.2000-7426.rela0138

[49] Boehlen FH Herzog W Maatouk I Saum K-U Brenner H Wild B. Treatment preferences of elderly patients with mental disorders. Z Gerontol Geriatr. (2016) 49:120–5. 10.1007/s00391-015-0908-x 26033574

[50] Kitchen KA McKibbin CL Wykes TL Lee AA Carrico CP McConnell KA. Depression treatment among rural older adults: preferences and factors influencing future service use. Clin Gerontol. (2013) 36:1–15. 10.1080/07317115.2013.767872 24409008 PMC3881270

[51] Da Silva PFR Blay SL. Prevalence and characteristics of outpatient psychotherapy use: a systematic review. J Nerv Ment Dis. (2010) 198:783–9. 10.1097/NMD.0b013e3181f97e1f 21048467

[52] Reavley NJ Morgan AJ Jorm AF. Development of scales to assess mental health literacy relating to recognition of and interventions for depression, anxiety disorders and schizophrenia/psychosis. Aust N Z J Psychiatry (2014) 48:61–9. 10.1177/0004867413491157 23744982

[53] Dunn KI Goldney RD Grande ED Taylor A. Quantification and examination of depression-related mental health literacy. J Eval Clin Pract. (2009) 15:650–3. 10.1111/j.1365-2753.2008.01067.x 19522721

[54] Kaneko Y Motohashi Y. Male gender and low education with poor mental health literacy: a population-based study. J Epidemiol. (2007) 17:114–9. 10.2188/jea.17.114 17641446 PMC7058472

[55] Mackenzie CS Gekoski WL Knox VJ. Age, gender, and the underutilization of mental health services: the influence of help-seeking attitudes. Aging Ment Health (2006) 10:574–82. 10.1080/13607860600641200 17050086

[56] Kessler RC Demler O Frank RG Olfson M Pincus HA Walters EE. Prevalence and treatment of mental disorders, 1990 to 2003. N Engl J Med. (2005) 352:2515–23. 10.1056/NEJMsa043266 15958807 PMC2847367

[57] Dunlop BW Kelley ME Mletzko TC Velasquez CM Craighead WE Mayberg HS. Depression beliefs, treatment preference, and outcomes in a randomized trial for major depressive disorder. J Psychiatr Res. (2012) 46:375–81. 10.1016/j.jpsychires.2011.11.003 22118808 PMC3288535

[58] Christensen H Griffiths KM Evans K e-Mental Health in Australia: Implications of the Internet and Related Technologies for Policy: ISC Disussion Paper No.3. Canberra, ACT: Commonwealth Department of Health and Ageing (2002).

[59] Karyotaki E Riper H Twisk J Hoogendoorn A Kleiboer A Mira A. Efficacy of self-guided internet-based cognitive behavioral therapy in the treatment of depressive symptoms: a meta-analysis of individual participant data. JAMA Psychiatry (2017) 74:351–9. 10.1001/jamapsychiatry.2017.0044 28241179

[60] Sikorski C Luppa M Kersting A König H-H Riedel-Heller SG. Computer-aided cognitive behavioral therapy for depression. Psychiatr Prax. (2011) 38:61–8. 10.1055/s-0030-1248575 20972949

[61] Richards D Richardson T. Computer-based psychological treatments for depression: a systematic review and meta-analysis. Clin Psychol Rev. (2012) 32:329–42. 10.1016/j.cpr.2012.02.004 22466510

[62] Stein J Röhr S Luck T Löbner M Riedel-Heller SG. Indication and evidence of internationally developed online coaches as intervention for mental illness - a meta-review. Psychiatr Prax. (2017) 45:7–15. 10.1055/s-0043-117050 28851002

[63] Rost T Stein J Löbner M Kersting A Luck-Sikorski C Riedel-Heller SG. User acceptance of computerized cognitive behavioral therapy for depression: systematic review. J Med Internet Res. (2017) 19:e309. 10.2196/jmir.7662 28903893 PMC5617907

[64] Riedel-Heller SG Gühne U Weinmann S Becker T. Psychosocial therapies in severe mental illness: DGPPN-S3-guideline: evidence, recommendations and challenges for mental health service research. Psychother Psychosom Med Psychol. (2012) 62:425–8. 10.1055/s-0032-1327676 23143829

[65] Riedel-Heller SG Gühne U Weinmann S Arnold K Ay E-S Becker T. Psychosocial interventions in severe mental illness: evidence and recommendations: psychoeducation, social skill training and exercise. Nervenarzt (2012) 83:847–54. 10.1007/s00115-011-3471-8 22729513

[66] Klein JP Gerlinger G Knaevelsrud C Bohus M Meisenzahl E Kersting A Internetbasierte interventionen in der behandlung psychischer storungen: uberblick, qualitatskriterien, perspektiven. Nervenarzt (2016) 87:1185–93. 10.1007/s00115-016-0217-7 27649987

[67] Dorow M Stein J Förster F Löbner M Franz M Günther R. Implementation of the internet-based self-management program “moodgym” in patients with depressive disorders in inpatient clinical settings - patient and expert perspectives. Psychiatr Prax. (2017). [Epub ahead of print]. 10.1055/s-0043-117049 28851000

[68] Batterham PJ Calear AL. Preferences for internet-based mental health interventions in an adult online sample: findings from an online community survey. JMIR Ment Health (2017) 4:e26. 10.2196/mental.7722 28666976 PMC5511366

[69] Statistisches Bundesamt IT-Nutzung (2015). Available online at: https://www.destatis.de/DE/ZahlenFakten/GesellschaftStaat/EinkommenKonsumLebensbedingungen/ITNutzung/ITNutzung.html. (Accessed June 28, 2016).

[70] Kruse RL Koopman RJ Wakefield BJ Wakefield DS Keplinger LE Canfield SM. Internet use by primary care patients: where is the digital divide? Fam Med. (2012) 44:342–7. 23027117

[71] Eichenberg C Wolters C Brähler E. The internet as a mental health advisor in Germany–results of a national survey. PLoS ONE (2013) 8:e79206. 10.1371/journal.pone.0079206 24278121 PMC3836792

[72] Löbner M Stein J Rost T Förster F Dorow M Keller J Innovative E-Health-ansätze für komorbide depressionen bei patienten mit adipositas: nutzungsakzeptanz aus patienten- und expertenperspektive. Psychiatr Prax. (2017) 44:286–95. 10.1055/s-0043-107471 28403502

[73] Crisp DA Griffiths KM. Participating in online mental health interventions: who is most likely to sign up and why? Depress Res Treat. (2014) 2014:790457. 10.1155/2014/790457 24804089 PMC3996960

[74] Donkin L Hickie IB Christensen H Naismith SL Neal B Cockayne NL. Sampling bias in an internet treatment trial for depression. Transl Psychiatry (2012) 2:e174. 10.1038/tp.2012.100 23092978 PMC3565809

[75] Warmerdam L van Straten A Twisk J Riper H Cuijpers P. Internet-based treatment for adults with depressive symptoms: randomized controlled trial. J Med Internet Res. (2008) 10:e44. 10.2196/jmir.1094 19033149 PMC2629364

[76] Høifødt RS Lillevoll KR Griffiths KM Wilsgaard T Eisemann M Waterloo K. The clinical effectiveness of web-based cognitive behavioral therapy with face-to-face therapist support for depressed primary care patients: randomized controlled trial. J Med Internet Res. (2013) 15:e153. 10.2196/jmir.2714 23916965 PMC3742404

[77] Mojtabai R Olfson M Mechanic D Perceived need and help-seeking in adults with mood, anxiety, or substance use disorders. Arch Gen Psychiatry (2002) 59:77–84. 10.1001/archpsyc.59.1.77 11779286

[78] Halmetoja CO Malmquist A Carlbring P Andersson G Experiences of internet-delivered cognitive behavior therapy for social anxiety disorder four years later: a qualitative study. Internet Interv. (2014) 1:158–63. 10.1016/j.invent.2014.08.001

[79] Wallin EEK Mattsson S Olsson EMG. The preference for internet-based psychological interventions by individuals without past or current use of mental health treatment delivered online: a survey study with mixed-methods analysis. JMIR Ment Health (2016) 3:e25. 10.2196/mental.5324 27302200 PMC4925931

[80] Klein B Cook S Preferences for e-mental health services amongst an online Australian sample. E-J Appl Psychol. (2010) 6:28–39. 10.7790/ejap.v6i1.184

[81] Apolinario-Hagen J Kemper J Sturmer C. Public acceptability of E-mental health treatment services for psychological problems: a scoping review. JMIR Ment Health (2017) 4:e10. 10.2196/mental.6186 28373153 PMC5394261

[82] Casey LM Joy A Clough BA. The impact of information on attitudes toward e-mental health services. Cyberpsychol Behav Soc Netw. (2013) 16:593–8. 10.1089/cyber.2012.0515 23679567

[83] Musiat P Goldstone P Tarrier N. Understanding the acceptability of e-mental health–attitudes and expectations towards computerised self-help treatments for mental health problems. BMC Psychiatry (2014) 14:109. 10.1186/1471-244X-14-109 24725765 PMC3999507

[84] Schomerus G Angermeyer MC Baumeister SE Stolzenburg S Link BG Phelan JC. An online intervention using information on the mental health-mental illness continuum to reduce stigma. Eur Psychiatry (2016) 32:21–7. 10.1016/j.eurpsy.2015.11.006 26802980

[85] Stolzenburg S Freitag S Evans-Lacko S Muehlan H Schmidt S Schomerus G. The stigma of mental illness as a barrier to self labeling as having a mental illness. J Nerv Ment Dis. (2017) 205:903–9. 10.1097/NMD.0000000000000756 29099405

[86] Casey LM Wright M-A Clough BA Comparison of perceived barriers and treatment preferences associated with internet-based and face-to-face psychological treatment of depression. Int J Cyber Behav Psychol Learn. (2014) 4:16–22. 10.4018/ijcbpl.2014100102

