# Supplementary material for: Preferences for Depression Treatment Including Internet-Based Interventions: Results From a Large Sample of Primary Care Patients
Source: Front Psychiatry. 2018 May 17;9:181. doi: 10.3389/fpsyt.2018.00181 (PMC5966543; doi:10.3389/fpsyt.2018.00181)
Supplement: Supplementary file 1 [file Data_Sheet_1.docx]

**Figure S1** Treatment preferences for depression in primary care


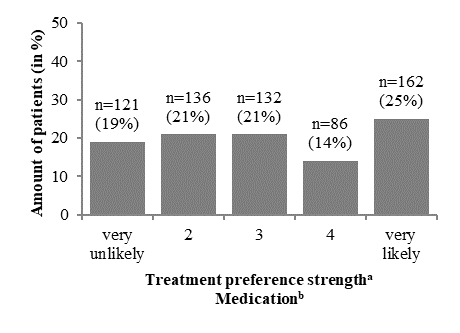

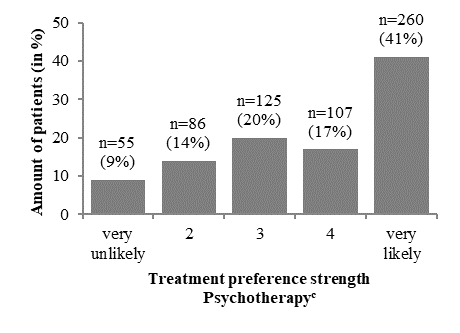

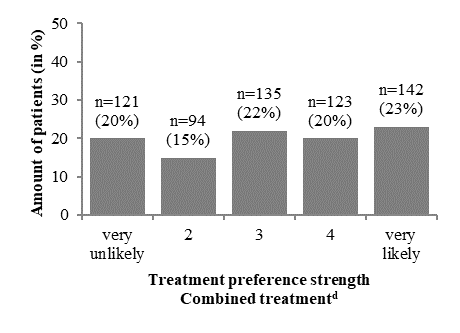

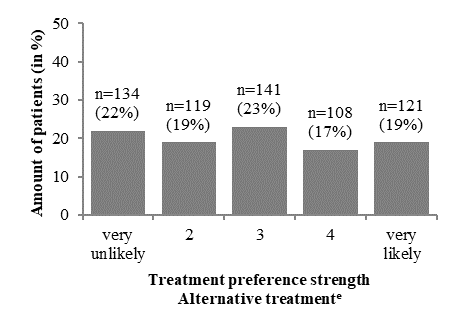

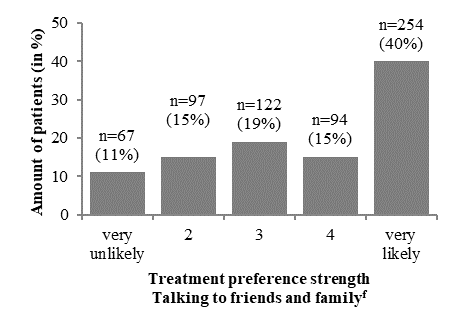

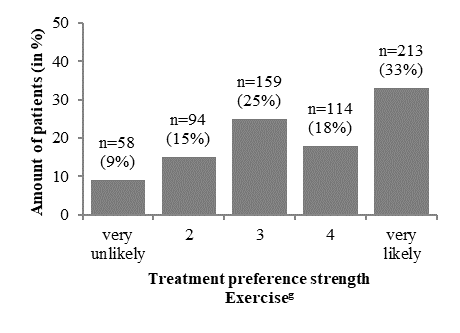

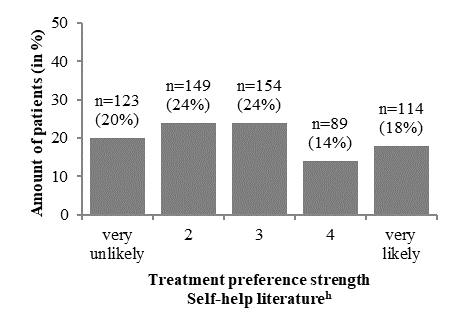

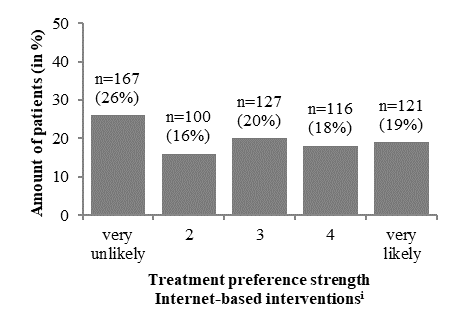


| ^a^ Response to the question: “To want extent would you consider [type of treatment option] in case of depression?”; ^b^ n = 637; ^c^ n = 633; ^d^ Combined treatment refers to medication and psychotherapy, n = 615; ^e^ Alternative treatment (e.g. alternative practitioners), n = 623; ^f^ n = 634; ^g^ n = 638; ^h^ n = 629; ^i^ n = 631 |
| --- |
